# Discrimination against Rural-to-Urban Migrants: The Role of the *Hukou* System in China

**DOI:** 10.1371/journal.pone.0046932

**Published:** 2012-11-05

**Authors:** Lei Kuang, Li Liu

**Affiliations:** School of Psychology, Beijing Normal University, Beijing, China; The University of South Wales, Australia

## Abstract

China's rural-urban dual society system is instituted by its unique *hukou* system. This system causes inequalities in social status between permanent urban and rural residents, and discrimination against rural-to-urban migrants is thus prevalent. A series of studies, based on system justification theory, sought to address the impact of the *hukou* system on the discrimination against rural-to-urban migrants. Study 1 showed that the justification of the *hukou* system could predict discrimination operationalized using a social distance measure. Study 2 found that priming of the proposed abolishment of the current *hukou* system led to reduced social distance. Study 3, using a recruiting scenario, further demonstrated that priming of the proposed abolishment of the system led to reduced discrimination in salary decision. Consistent with our predictions, discrimination against rural-to-urban migrants could be triggered by justifying the current *hukou* system, while priming of the abolishment of the system serves to decrease discrimination. The present research thereby sheds light on China's reform of its *hukou* system to achieve social justice and equality from a psychological perspective.

## Introduction

Discrimination against marginalized groups is a global psychosocial phenomenon, but it retains its local character in a given social, cultural, and economic context. From a global perspective, racial minorities, people living with HIV/AIDS, the disabled, the obese, homosexuals, and the physically unattractive are all examples of targets of discrimination [1]–[4]. From a local perspective, discrimination against rural-to-urban migrants, due to the unique *hukou* system, is salient in China [5]–[7]. However, the role of social institutions, such as the *hukou* system, in discrimination is still subject to scientific debate. The aim of the current study is to explore the impact of the *hukou* system and its reform on discrimination against rural-to-urban migrants.

The *hukou* system, modeled from the Soviet *propiska* (internal passport), translates literally as the “household registration system” but differs substantially from other systems of household registration [8]. Beginning in the 1950s, the Chinese government officially promulgated its *hukou* system to differentiate residential groups [9]–[10]. The *hukou* system was seen as an indispensable feature of Chinese socialist economic planning and was designed to forestall rural-to-urban migration [11]. Every citizen is required to register at their permanent residence, as registration under the *hukou* system is the principal means of establishing one's official status in China [12]. One's *hukou* status is inherited from one's mother and thus is predetermined. While initially conceived as an instrument for internal migration control, the *hukou* system was soon transformed into a social institution dividing Chinese society into spatial hierarchies [13]. There is a dual classification in a person's *hukou*. The first is one's residence, which is commonly referred to as rural/urban area; and the second is one's socio-economic eligibility, which is commonly referred to as agricultural/non- agricultural category. Individuals registered under the agricultural category depended mainly on their own labor and the fluctuating harvests for survival; individuals registered under the non-agricultural category, on the other hand, were entitled to a “cradle-to-grave” welfare package provided by the government. As a result, urban residents were seen as superior to rural residents in terms of socio-economic status.

Since its shift towards economic liberalism in the 1980s, China has initiated a variety of reforms to the *hukou* system. Although the *hukou* system is no longer used to prevent rural-to-urban mobility, Chinese society can still be divided into an agricultural segment and a non-agricultural one. This division remains crucial in determining people's opportunities. There are a few channels by which one can convert from an agricultural to a non-agricultural *hukou* status, for instance, recruitment for enrollment in an institution of higher education. The further reform of the *hukou* system, as a controversial topic, has been widely discussed among policymakers and in the general public [8]. The abolishment of the agricultural and non-agricultural *hukou* distinction has thus been tested in a few places as a pilot schema. However, the *hukou* system in general remains potent and continues to function as a key institution, perpetuating China's rural-urban disparity [8].

In China, The term “rural-to-urban migrants” refers to farmers-turned-workers who move from rural to urban areas for jobs and better lives without obtaining permanent urban residency [6]. China's liberal economic reform has resulted in rapid economic growth in cities, which in turn creates millions of job vacancies. On the other hand, this economic growth significantly decreased arable land in rural areas, which in turn produced millions of surplus rural laborers [7]. As a result, the restriction on rural-to-urban mobility has been relaxed. According to the Ministry of Human Resources and Social Security of China, in 2010, approximately 153.35 million rural residents had migrated into cities [14]. However, the *hukou* system still denies the migrants permanent urban residency rights and many of the associated social benefits. As a consequence, they must move back and forth between the cities, where they work and temporarily reside, and their home villages, where they belong permanently. In this sense, it is the unequal system that disadvantages rural-to-urban migrants in Chinese society.

Despite their indispensable roles in Chinese economic growth, migrants, or “outsiders”, are segregated from urban host populations. They are considered “backward”, portrayed negatively by indigenous urban residents, and maltreated at work. They must work so-called “3D jobs” (dangerous, dirty and demeaning jobs) [15], that permanent urban residents generally find inferior and undesirable [16]–[17]. As such, an agricultural *hukou* functions as a maligned social label. People normally infer lower socioeconomic status and often negative traits for individuals with an agricultural *hukou*. Permanent urban residents have little desire for contact with the migrants unless they must [18]. Social distance between the two groups has thus gradually increased [18]–[23]. Discrimination against rural-to-urban migrants is an irrefutable indicator of the strained relations between the two social groups. In short, the literature shows a high level of discrimination against rural-to-urban migrants. However, the impact of *hukou* system on the discrimination remains underexplored by psychologists, as does the answer for how to reduce it through effective political reform.

An unequal social institution in psychological literature is related to intergroup discrimination. The issue of intergroup discrimination has drawn great attention from the theories of social identity, social dominance, and system justification. However, they approach the issue from different perspectives. Social identity theory emphases the role of such cognitive processes as social categorization and social comparison in creating intergroup discrimination. It is evident that simply categorizing individuals into ingroup and outgroup is sufficient to generate intergroup relational problems [24]–[25]. Social dominance theory is a theory of intergroup relations that focuses on the maintenance and stability of group-based social hierarchy. It emphases the dynamics of power struggle between the members of dominant groups and subordinate groups [26].

The present research is based on system justification theory because *hukou* can be considered as a social system or an institution that promoting the status quo such that people with a non- agricultural *hukou* usually enjoy better social privileges than those with an agricultural *hukou*. System justification is defined as perceptions of the fairness, legitimacy, and justifiability of a social institution [27]. System justification theory postulates that people in general are motivated to defend, justify, and bolster aspects of the status quo, including existing political institutions and social arrangements [28]. The empirical researches on system justification have shown that stereotyping and prejudice are related to attitudes about social and political systems. Specifically, as the tendency of perceiving political or economic inequality to be fair, legitimate, and necessary increase, members of high-status groups will exhibit increased ingroup favoritism [29]–[30]. Increased prejudice can be predicted by justifying and rationalizing existing inequality, because stereotypes and other social judgments might be a kind of ideological support for the prevailing social system [31]. Similarly, researchers have found that people who consider the unequal system or hierarchy to be good are more likely to derogate members of disadvantaged groups, such as African Americans, homosexuals and the obese [32]–[35]. Overall, the justification of an unequal social system, which serves to support the imbalanced arrangement, endorses discrimination against members of low-status groups.

Although system justification motivation is generally associated with prejudice and denial of social change, it is possible to harness this motivation in a constructive manner. Experiments by Kay, Jimenez, and Jost suggest that, when regime change seems highly probable, people begin to rationalize the new arrangements almost immediately [36]. They are more willing to embrace social change, instead of resisting, when the change seems inevitable. It can be inferred that implementing a new social institutions that emphasizes social integration and equality will make people embrace the reform, which might be associated with decreased prejudice and discrimination. The evidence about racism in America supports our inference. For instance, landmark legislative decisions in America (e.g., the Supreme Court ruling on school desegregation in 1954 and the Civil Rights laws of the early 1960s) have made race-based discrimination illegal [37]. Then in the subsequent 50 years, overt expression of discrimination against African-Americans appears to have decreased [38]–[40]. These findings are also consistent with Perlstein's assertion that the reform of a social system directly reduces discrimination [41]. Overall, it is evident that intergroup discrimination is affected by political actions, whether the actions are imagined or real [37]. Thus, it is reasonable to predict that reform of the current *hukou* system can help to reduce discrimination against rural-to-urban migrants in China.

In sum, the evidence suggests that (a) the justification of an unequal system is associated with stereotyping and prejudice towards disadvantaged groups; (b) an inevitable political reform will be immediately rationalized by people; and (c) political reform promoting social integration can reduce discrimination. Based on these previous findings, we raise a possibility: justification of the unequal *hukou* system and the reform of system could have a great impact on discrimination against rural-to-urban migrants in China. Because two types of potential policy, the abolishment versus preservation of the current *hukou* system have been widely circulated in public discourse, reading a related article can raise the accessibility of one type of the policy. We specifically predict that 1) justification of the current *hukou* system is associated with discrimination against rural-to-urban migrants and 2) priming the preservation of the current system could potentially trigger discrimination, while priming abolishment of the unequal system would serve to decrease the discrimination.

For the current research, we used both correlational and experimental designs to examine the extent to which the *hukou* system impacts discrimination against rural-to-urban migrants. Three studies were conducted to test our predictions. Study 1 was designed to investigate the association between justification and discrimination in the current *hukou* system. In Study 2, the causal effect of the *hukou* system on discrimination was examined by experimentally manipulating the *hukou* system's reform. We expected that discrimination would decrease when the *hukou* system was primed to be abolished and vice versa. In Study 3, using an employment recruiting scenario, we explored whether discrimination in such a context could be reduced by priming abolishment of the *hukou* system, thus testing the generality of our findings.

## Study 1

### Methods

#### Ethics Statement

The study was reviewed and approved by the Committee of Protection of Subjects at Beijing Normal University. All participants provided written informed consent before the study, and they were fully debriefed at the end of the research according to the established guidelines of the committee. This procedure was followed in Studies 2 and 3 as well.

#### Participants

In Study 1, 157 undergraduate students (102 females, 54 males, 1 did not report gender) participated. All of the participants were registered in the non-agricultural *hukou* category when enrolled in the study. Among them, 52.2% reported that they were originally registered in the non-agricultural *hukou* category prior to their university enrollment, and 47.8% reported that they were originally registered in the agricultural *hukou* category prior to their university enrollment.

#### Measures and procedures

A questionnaire was administrated to all participants. The questionnaire consisted of three parts, and the items were all measured on a 7-point scale, ranging from *1* (*completely disagree*) to *7* (*completely agree*), except in part 3.

Part 1 contained a brief introduction to the current *hukou* system in China and an 8-item *hukou* system justification scale. Adapted from Kay and Jost's items, the scale was used to measure perceptions of the fairness, legitimacy, and justifiability of the *hukou* system in China (for example, “In general, the *hukou* system in China operates as it should”, and “The Chinese *hukou* system needs to be radically restructured” (reverse-scored)) [42]. Participants were asked to indicate their degree of agreement or disagreement with each item. One of the 8 items was excluded during analysis to obtain satisfactory reliability (*α* = .820) (the dropped item was “Most policies related to *hukou* serve the greater good”). Higher scores indicated increased levels of system justification.

In part 2, a 9-item adapted Bogardus social distance scale was used to measure discrimination against rural-to-urban migrants (for instance, “I prefer to avoid to contact with rural-to-urban migrants”) [43]. Higher scores represented a greater desire to distance oneself from rural-to-urban migrants (α = .912).

In part 3, the participants were required to complete the Responding Desirably on Attitudes and Options Scale (RD-16) to control for socially desirable responding (for example, “I feel that I am better off than my parents were at my age”) [44]–[45]. The scale consisted of 16 items that were all measured on a 2-point scale (*1 = disagree, 2 = agree*). Higher scores were coded to indicate a higher level of social desirability (α = .701).

### Results and Discussion

The descriptive analysis and correlations between the 3 continuous variables are presented in Table 1 below. As Table 1 shows, both the justification of the *hukou* system and social desirability are significantly correlated with discrimination.

**Table 1:** Descriptive analysis and correlations in Study 1.

Variable	*N*	*M*	*SD*	1	2	3
1 justification of the *hukou* sys**tem**	157	3.29	1.06	—		
2 discrimination	157	2.77	0.99	0.22**	—	
3 social desirability	157	11.86	2.72	0.06	0.19*	—

* *p*<0.05;

** *p*<0.01.

We first conducted an one way ANOVA (original *hukou* category: agricultural vs. non-agricultural) to examine the effect of participants' original *hukou* category on justification of the *hukou* system. The result showed that there were significant difference in terms of system justification scores between participants who were originally registered in the agricultural vs. non-agricultural categories (*F* (1, 156) = 4.723, *p* = .031). The participants originally categorized as non-agricultural *hukou* citizens considered the current *hukou* system more legitimate and fair (*M* = 3.46, *SD* = 1.06) than those who were originally agricultural *hukou* citizens (*M* = 3.10, *SD* = 1.03).

Subsequently, we regressed discrimination onto justification of the *hukou* system, original *hukou* category, and justification×original *hukou* category interaction, to examine whether participants' justifications for the *hukou* system predicted their discrimination, regardless of their original *hukou* category. Before analysis, those who originally registered in the agricultural *hukou* category were coded “0”, while those registered in the non-agricultural *hukou* category were coded “1”. The hierarchical regression revealed that, after statistically controlling for the effects of social desirability, the main effects of justification of the *hukou* system (*β* = .171, t = 2.320, *p*<.05) and original *hukou* category (*β* = .339, t = 4.602, *p*<.001) were both significant. As predicted, discrimination against rural-to-urban migrants at least partially originated from justification of the *hukou* system. Specifically, greater justification of the unequal *hukou* system by participants predicted stronger discrimination by those same individuals. In addition, discrimination against rural-to-urban migrants was associated with the participants' original *hukou* category. However, an insignificant justification×*hukou* type interaction (*β* = .033, t = .455, *p* = .650) was found, indicating that the participants' original *hukou* category did not moderate the association between justification of the *hukou* system and discrimination. Overall, no matter what their original *hukou* categories were, participants' discrimination against rural-to-urban migrants could be significantly predicted by their justification of the *hukou* system.

These results are consistent with the vast literature indicating that justification of an unequal social system is positively associated with discrimination [29]–[31]. The current study extended previous research by first clarifying the relationship between system justification and discrimination in the context of China's unique *hukou* system. This study suggests that to increase Chinese social cohesion, the reform of the unequal social institution is badly needed. However, it remains unclear whether discrimination against rural-to-urban migrants could be reduced through the reform of the *hukou* system. This is what we focused on in Studies 2 and 3.

## Study 2

Study 1 revealed an association between the unequal *hukou* system and discrimination against rural-to-urban migrants. However, the correlational design of Study 1 did not allow for the investigation of the causal impact of the *hukou* system on discrimination. More importantly, it is worth exploring whether discrimination against migrants could be directly reduced through reform of the *hukou* system. Therefore, an experimental manipulation of the reform of the *hukou* system was used in Study 2 to investigate the issue. We hypothesized that anticipated abolishment of the *hukou* system, primed by mock articles, would decrease discrimination against migrants.

### Methods

#### Participants

Undergraduates in Beijing voluntarily participated in the study. After removal of 4 students' data by the manipulation check, the data from 54 participants (46 males, 8 females) were entered. All of the participants were registered in the non-agricultural *hukou* category when they participated in the study. Among them, 35 participants (64.8% of the sample) reported that they were originally registered in the non-agricultural *hukou* category prior to their university enrollment, while the rest of the sample was originally registered in the agricultural *hukou* category. The participants were randomly assigned to one of two between-subjects priming conditions, abolishing versus preserving the current system.

#### Materials and procedures

The reform of the *hukou* system was primed by one of two mock articles. In the abolishing condition, participants read the article indicating that “the Chinese government announces that the agricultural and non-agricultural *hukou* distinction is expected to be eliminated in 2016” (Figure S1 & Text S1). In the preserving condition, the article indicating “the Chinese government announces that the reform of agricultural and non-agricultural *hukou* distinction needs more investigation and that the current *hukou* system will be retained over a long period of time” (Figure S2 & Text S2).

After reading, the participants were asked to complete a 3-item manipulation check. The first two items were aimed at assessing the difficulty of the reading (“Do you feel the article was easy to understand?” and “Do you feel the expression of the article was clear?”). The third item checked participants' understanding of the article (“Which of the following sentences best summarizes the article?”). Only participants who felt that the priming material was not difficult to read and chose the correct summarizing sentence during the manipulation check were included in further analysis.

In the following steps, the participants were asked to complete the adapted Bogardus social distance scale (*α* = .891) and the RD-16 scale (*α* = .598) [43]–[45], which were identical to the ones used in Study 1, to indicate their discrimination against the migrants and their social desirability, respectively.

### Results and Discussion

We conducted a 2 (priming: abolishment vs. preservation)×2 (original *hukou* category: agricultural vs. non-agricultural) ANOVA controlling for social desirability to examine the effects of priming of the *hukou* system's abolishment or preservation and the participants' original *hukou* category on discrimination. The main effects of priming (*F* (1, 52) = 5.416, *p*<.05, *η_p_^2^* = .100) and original *hukou* category (*F* (1, 52) = 15.862, *p*<.001, *η_p_^2^* = .245) were both significant. As predicted, after statistically controlling for the effects of social desirability, the discrimination reported in the abolishing condition was less than that in the preserving condition (Table 2). In addition, the participants originally categorized as agricultural *hukou* citizens reported significantly less discrimination than those who were originally non-agricultural *hukou* citizens (Table 2). Again, no significant interaction was found between priming of the *hukou* system's abolishment or preservation and the original *hukou* category (*F* (1, 52) = 1.370, *p* = .248, *η_p_^2^* = .027). The results suggested that regardless of their *hukou* categories, the participants reported less discrimination when the unequal *hukou* system was primed to be abolished than when it was primed to be preserved.

**Table 2:** Main effects of the Between-subjects factor in Study 2.

Variable		*M ± SD*	df	*F*	*p*	*η_p_^2^*
Priming of the *hukou* sys**tem**	abolishing	2.72±0.93	1	5.416	0.024	0.100
	preserving	3.17±0.83	—	—	—	—
Original hukou category	agricultural	2.37±0.74	1	15.862	0.000	0.245
	non-agricultural	3.27±0.83	—	—	—	—
Priming×Original hukou category		—	1	1.370	0.248	0.027

Consistent with our prediction, when priming for the abolishment of the *hukou* system, the participants showed lower levels of discrimination against migrants. This result provided additional experimental support for previous work asserting that intergroup discrimination can be affected by political reformation, whether the action was imagined or real [37], [41].

In addition, the effect of participants' *hukou* categories prior to university enrollment on discrimination in both Studies 1 and 2 was striking but reasonable. To some extent, the university students who were originally registered in the agricultural *hukou* category at birth but had moved into the non-agricultural *hukou* category due to their university enrollment are still “migrants from the countryside”. As such, their discrimination against rural-to-urban migrants is lower than that of individuals who have been living in cities since birth. Regardless of where participants are from, however, their discrimination against rural-to-urban migrants can be reduced by priming the abolishment of the current *hukou* system, further confirming our hypothesis.

## Study 3

The evidence for the causal effect of the *hukou* system's reform on discrimination was compelling in Study 2. However, Studies 1 and 2 both used the index of social distance for the assessment of discrimination. Is it possible to replicate the findings in a contextualized situation, using a different indicator of discrimination? The objective of Study 3 was to further confirm the findings of Study 2 using an employment recruiting scenario, thereby generalizing the findings.

In Study 3, the hiring preference for two job candidates (a permanent urban resident vs. a rural-to-urban migrant) was used to indicate the levels of discrimination. This decision was based on the rationale that unwillingness to accept members of an outgroup as colleagues is a symbol of intergroup discrimination [37], [46]. We expected the difference in hiring preference for the two candidates to be smaller when the *hukou* system was primed to be abolished than when the system was primed to be preserved.

### Methods

#### Participants

Undergraduates in Beijing voluntarily participated in Study 3. After removal of 7 students' data by the manipulation check, the data from 134 participants (101 males, 33 females) were entered. All of the participants were registered in the non-agricultural *hukou* category when they participated in the study. Among the participants, 61.2% reported that they were originally registered in the non-agricultural *hukou* category prior to their university enrollment, while 38.8% reported that they were registered in the non-agricultural *hukou* category prior to their university enrollment. All of the participants were randomly assigned to a priming condition (abolishing or preserving the system).

#### Materials and procedures

The study used a 2 (the priming of the *hukou* system: abolishing the *hukou* system vs. preserving the *hukou* system)×2 (original *hukou* category: agricultural vs. non-agricultural)×2 (job candidate type: a permanent urban resident vs. a rural-to-urban migrant) mixed design. The first two are between-subjects factors, while the last one is within-subjects factor. The participants were first asked to read one of the priming materials used in Study 2. The manipulation check was also the same as in Study 2.

After the priming, the participants were asked to read a recruitment notice following the Goldberg paradigm [47], describing a job vacancy for a customer service staff member in an insurance company. Within the recruitment notice, requirements and responsibilities of the job were introduced in detail (see supplementary materials). The participants, who were asked to act as a HR recruitment specialist of the company in the situation, needed to read and evaluate resumes of the two candidates who were competing for the position. Four sections were included in each resume: career objective, self-assessment, work experience and educational background (Text S3). The two resumes were set equal in objective qualifications, except for their *hukou* identifications: one was a permanent urban resident holding a non-agricultural *hukou* status, while the other was a rural-to-urban migrant holding an agricultural *hukou* status.

After reading each resume, the participants completed a questionnaire consisting of four parts (Text S4). The first part was a 4-item manipulation check to make sure that participants read the resumes carefully (for example, fill in the last 4 digits of the candidate's mobile number). The second part was designed to indicate the participants' perceptions of two candidates' qualifications, including the candidates' prior work experience (for example, “the strength of past experience”) and the overall impression of the candidates. The third part was designed to reveal participants' blatant and explicit discrimination operationalized using a hire decision measure (“If you were offering the job, how likely is it that you would hire the applicant?”). The fourth part was designed to reveal participants' subtle and indirect discrimination operationalized using a salary entitlement measure (“If the applicant were hired, how much do you think the person should earn (Yuan)?”. All of the measures were completed using a 7-point Likert-type scale. For the salary entitlement measure, Yuan, the Chinese currency unit was labeled in the 7-point Likert scale, that is, 1 = 1,600 Yuan, 2 = 2,000 Yuan, 3 = 2,400 Yuan, 4 = 2,800 Yuan, 5 = 3,200 Yuan, 6 = 3,600 Yuan, and 7 = 4,000 Yuan. The Cronbach's alphas were both tolerable (*α*_urban_ = .907; *α*_migrant_ = .885). The participants also completed the RD-16 scale (*α* = .646) used in Studies 1 and 2 [44]–[45].

### Results and Discussion

A 2 (prime: abolishment vs. preservation)×2 (original *hukou* category: agricultural vs. non-agricultural)×2 (job candidate type: urban resident vs. rural-to-urban migrant) Repeated Measure controlling for social desirability was conducted. First, we tested the main effects of the within-subjects factor (job candidate type). After statistically controlling for the effect of social desirability, there was no significant difference in the evaluation of prior work experience or overall impression between the two candidates (Table 3). It suggested, participants believed that the two candidates were “equal” in qualifications, which was consistent with our expectations. In addition, the results revealed that the main effect of job candidate type was not significant for either the likelihood of hire or salary offer (Table 3).

**Table 3:** Main effects of the within-subjects factor (job candidate type) in Study 3.

Evaluation dimension	Urban candidate (*M ± SD*)	Rural-to-urban candidate (*M ± SD*)	df	*F*	*p*	*η_p_^2^*
prior wor**k experience**	5.16±0.69	4.99±0.67	1	0.096	0.758	0.001
overall impression	5.28±0.85	5.17±0.84	1	1.817	0.180	0.014
likelihood of hire	5.25±1.03	4.91±0.98	1	1.040	0.310	0.008
salary offer	3.66±1.40	3.42±1.31	1	1.640	0.203	0.013

Furthermore, two between-subjects factors (the priming of the *hukou* system's status and the participants' original *hukou* categories) were included in the repeated measures to test whether they would influence the difference of the likelihood of hire for the two candidates. The results revealed that neither the priming nor the participants' original *hukou* categories had significant effects on differences in the likelihood of hire. The interaction effect of these two factors was also insignificant (Tables 4 & 5). In other words, regardless of the *hukou* system's reform status and the participants' original *hukou* categories, the difference in likelihood of hire for the two candidates did not vary significantly.

**Table 4:** Descriptive analysis in Study 3.

Candidate	Priming of the hukou system	Original hukou category	Likelihood of hire (M ± SD)	Salary offer (M ± SD)
Rural-to-urban migrant	Abolishing	agricultural	5.08±1.03	4.00±1.22
		non-agricultural	4.77±0.94	3.40±1.38
	Preserving	agricultural	5.07±1.10	3.41±1.31
		non-agricultural	4.83±0.92	3.14±1.26
Urban resident	Abolishing	agricultural	5.16±1.21	4.00±1.41
		non-agricultural	5.26±0.92	3.51±1.42
	Preserving	agricultural	5.56±1.15	3.78±1.40
		non-agricultural	5.11±0.91	3.52±1.39

**Table 5:** Effects of the between-subjects factors in Study 3.

Dependent variable	Factor	df	*F*	*p*	*η_p_^2^*
Likelihood of hire	Priming of the *hukou* system	1	0.924	0.338	0.007
	Original hukou category	1	3.063	0.082	0.024
	Priming×Original hukou category	1	2.512	0.115	0.019
Salary offer	Priming of the *hukou* system	1	7.107	0.009	0.053
	Original hukou category	1	6.735	0.011	0.050
	Priming×Original hukou category	1	0.126	0.723	0.001

**Figure 1:**
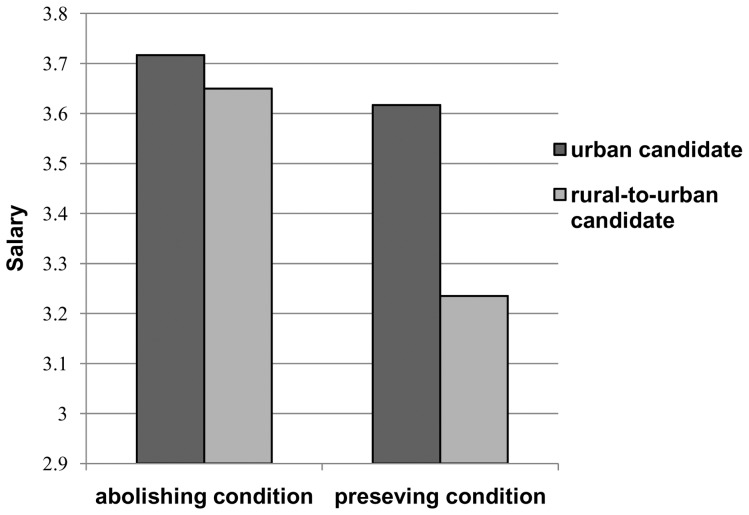
The impact of *hukou* system reform on salary. The priming of the *hukou* system's status significantly influenced the salary prospects of the two candidates. Specifically, the difference between the two candidates' potential salaries in the abolishing condition was smaller than the difference in the preserving condition.

However, as predicted, the priming of the *hukou* system's status significantly influenced the salary prospects of the two candidates (*F*(1, 127) = 7.107, *p*<.01, *η* ^2^ = .053, Tables 4 & 5). Specifically, the difference between the two candidates' potential salaries in the abolishing condition was smaller than the difference in the preserving condition (Figure 1). In other words, our hypothesis was partially supported by the effect of priming for the *hukou* system's abolishment on salary in this study. The main effect of original *hukou* category on the salary difference was also significant (*F* (1, 127) = 6.735, *p*<.05, *η_p_^2^* = .050, Tables 4 & 5). This effect was consistent with the findings of Studies 1 and 2. However, the analysis failed to find a significant interaction between *hukou* system reform status and original *hukou* category (*F* (1, 127) = .126, *p* = .723, *η_p_^2^* = .001, Tables 4 & 5). This result indicated that for the participants originally registered in either the non-agricultural or agricultural *hukou* categories, the difference in salary offers to the two candidates could be predicted by the priming for *hukou* system reform.

The most important finding of this study is that the difference between the two candidates' salaries after primed abolishing the *hukou* system was smaller than the salary difference under the current *hukou* system. In other words, priming abolishment of the *hukou* system reduced differences in proposed salaries between the two candidates. These findings provide further evidence that reform of the *hukou* system had an impact on discrimination against migrants. Previous research shows that disadvantaged groups are often rated negatively and offered less entitlement in occupational contexts [48]–[49]. However, with the improvement of social institution and norms, discrimination against disadvantaged groups has declined in the past few decades [50]–[51]. Consistent with these studies, the implication of the findings from our study is compelling; when primed preserving the current *hukou* system, the system itself gave unfair treatment to rural-to-urban migrants, and the migrants were belittled as a disadvantaged social group. However, in the case of *hukou* abolishment, system reform would promote equal treatment for rural-to-urban migrants and permanent urban residents.

Interestingly, the impact of *hukou* system's reform on the overt expression of discrimination against the migrants, indicated by the likelihood of hire, was not found in this study. It suggested that blatant or explicit discrimination displaying in a hiring decision was probably suppressed because the Chinese government has recently promulgated regulations against blatant discrimination [7]. People then turn to choose more subtle and indirect form to express their discrimination because it provides a cover, and protects a sense of egalitarianism and a non-prejudiced self-image [52].

## General Discussion

Overall, the findings of the three studies support our hypotheses; the Chinese *hukou* system is an institutional cause of discrimination against rural-to-urban migrants, while the priming abolishment of such a system serves to decrease discrimination. In Study 1, we showed that justification of the unequal *hukou* system is positively associated with discrimination operationalized as social distance, while in Study 2, we examined the effects of the *hukou* system's reform on discrimination. The results showed that social distance could be reduced when the system was primed to be abolished. In Study 3, we tested the generality of our findings and investigated whether the results of Study 2 could be replicated in an employment recruiting scenario. We found evidence that priming abolishment of the *hukou* system could reduce subtle discrimination operationalized as salary entitlement.

The current research demonstrates that system justification theory developed in western culture can be exported and tested in a Chinese cultural context. The rationale of the theory can be applied to explain and predict intergroup relation problems in China. At the same time, focusing on the impacts of the justification of China's unique *hukou* system, and its highly probable reform, on the discrimination against rural-to-urban migrants, the findings reported in the current research provides additional support for system justification theory.

System justification theory has gained its currency due to its critical and innovative contributions to intergroup relations research [53]. For many years, system justification research is much concerned with social control. That is, basic proposition of system justification theory is that people are motivated to defend, bolster and justify social, economic and political institutions and arrangements even when opportunities to rectify injustice are available [28]–[29]. At the same time, with regard to the prospect for social change, limited research suggests that system justification motivation leads people to either resist or embrace social change [28]. However, an important limitation in system justification literature is lack of empirical research on the effect of social shift on intergroup relations. To solve the limitation, the current research approaches the issue from a socially highly relevant context of the *hukou* system reform in China. It takes advantage of the unique historical opportunity of dramatic social transition in China to experimentally investigate the effect of a perceived *hukou* system shift on the discrimination against rural-to-urban migrants. It thereby establishes a theoretical connection between (a) people engage in anticipatory rationalization of likely sociopolitical outcomes [36] and (b) stereotyping and prejudice are related to attitudes about sociopolitical systems in system justification research [31]. In doing so, system justification is no longer restrict itself to serve to justify the status quo and bolster the legitimacy of the existing social order, but sheds light on social psychological consequences of sociopolitical transition. Indeed, the findings of the current research imply that framing proposed social change as “system-sanctioned” (i.e, the implementation of *hukou* system reform is seen as inevitable in this research) would lead to decrease the discrimination against disadvantaged groups. It thus provides very strong support for Jost and Hamilton's theoretical argument that “any effective attempt to ameliorate prejudice must take into account its unmistakable societal origin and lead ultimately to an unraveling of familiar justifications” [54].

From a practical perspective, we hope that the current research can bring psychological enlightenment to the compelling social issue of the *hukou* system in China. To the best of our knowledge, the current research is the first empirical study investigating the impact of the Chinese *hukou* system on intergroup relations. Sociologists and economists have already provided solid evidence that the current *hukou* system plays an important role in the allocation of economic resources, educational opportunities and other welfare benefits for rural-to-urban migrants [55]–[58]. Such inequalities have created many social problems, such as the rural-urban social division [7], [59]. However, it is still unclear whether the *hukou* system shapes individuals' attitude and behavior towards disadvantaged outgroups. Our research constitutes an initial step towards answering this question from a psychological perspective. The findings are promising, suggesting that if the current *hukou* system were to be abolished, discrimination against rural-to-urban migrants may be reduced. Meanwhile, it can be inferred from the findings that the rationalization of political reform and a new social institution is crucial in order for the government to eliminate inequality between different social groups and promote social inclusion.

## Supporting Information

Figure S1 **Priming material of the abolishing condition in Studies 2&3.** The article read by participants in the abolishing condition indicates that “the Chinese government announces that the agricultural and non-agricultural *hukou* distinction is expected to be eliminated in 2016”. It can raise the accessibility of the policy about abolishment of the current *hukou* system.
(DOCX)

Figure S2 **Priming material of the preserving condition in Studies 2&3.** The article read by participants in the preserving condition indicates that “the Chinese government announces that the reform of agricultural and non-agricultural *hukou* distinction needs more investigation and that the current *hukou* system will be retained over a long period of time”. It can raise the accessibility of the policy about preservation of the current *hukou* system.
(DOCX)

Text S1 **Priming material of the abolishing condition in English.** The English version of Figure S1.
(DOCX)

Text S2 **Priming material of the preserving condition in English.** The English version of Figure S2.
(DOCX)

Text S3 **Recruitment notice and resumes of the candidates.** These are materials used in Study 3. Within the recruitment notice, requirements and responsibilities of a vacant job are introduced. And the resumes of two candidates, which respectively contains four sections, are showed after the notice.
(DOCX)

Text S4 **Questionnaire used in the recruiting scenario.** In the recruiting scenario of Study 3, participants evaluate each candidate on this questionnaire.
(DOCX)
