# Supplementary figures and images for: Discrimination against Rural-to-Urban Migrants: The Role of the Hukou System in China
Source: PLoS One. 2012 Nov 5;7(11):e46932. doi: 10.1371/journal.pone.0046932 (PMC3489849; doi:10.1371/journal.pone.0046932)

**Figure S1** Priming material of the abolishing condition in Studies 2&3


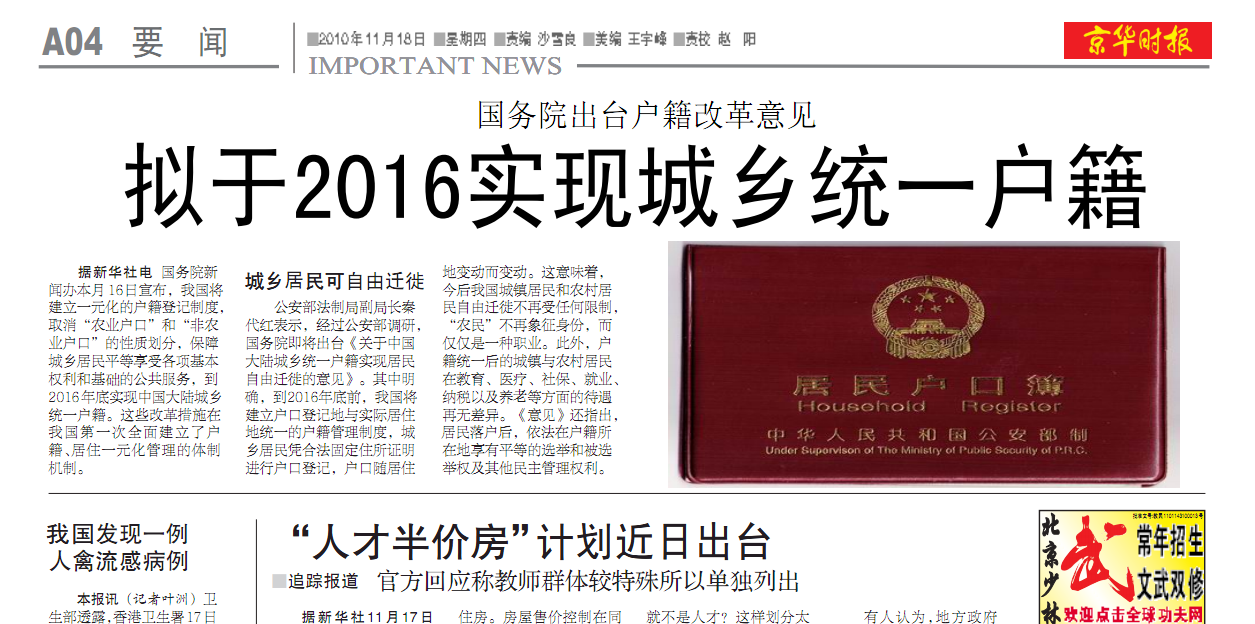

Supplement: Figure S1 — Priming material of the abolishing condition in Studies 2&3. The article read by participants in the abolishing condition indicates that “the Chinese government announces that the agricultural and non-agricultural hukou distinction is expected to be eliminated in 2016”. It can raise the accessibility of the policy about abolishment of the current hukou system. (DOCX) [file pone.0046932.s001.docx]

**Figure S2** Priming material of the preserving condition in Studies 2&3


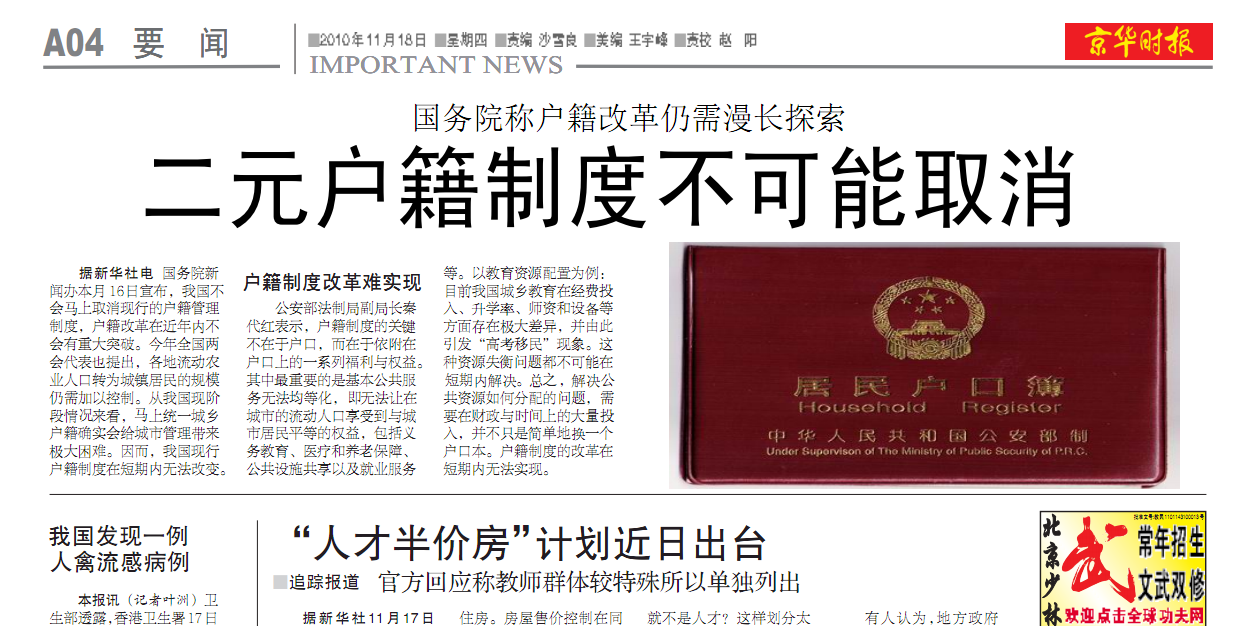

Supplement: Figure S2 — Priming material of the preserving condition in Studies 2&3. The article read by participants in the preserving condition indicates that “the Chinese government announces that the reform of agricultural and non-agricultural hukou distinction needs more investigation and that the current hukou system will be retained over a long period of time”. It can raise the accessibility of the policy about preservation of the current hukou system. (DOCX) [file pone.0046932.s002.docx]
