# Supplementary material for: Discrimination against Rural-to-Urban Migrants: The Role of the Hukou System in China
Source: PLoS One. 2012 Nov 5;7(11):e46932. doi: 10.1371/journal.pone.0046932 (PMC3489849; doi:10.1371/journal.pone.0046932)
Supplement: Text S1 — Priming material of the abolishing condition in English. The English version of Figure S1. (DOCX) [file pone.0046932.s003.docx]

**Text S1** Priming material of the abolishing condition in English

the Chinese government announces that

**the agricultural and non-agricultural *hukou* distinction is expected to be eliminated in 2016**

Xinhua News Agency news: The Information Office of the State Council announces in November 16^th^ that the Chinese Government will establish a unified household registration system and eliminate the agricultural and non-agricultural *hukou* distinction in 2016. This measure will ensure that urban residents and rural residents share fundamental right and welfare equally, which means that China will fully establish a hukou-inhabitancy unified management mechanism for the first time.

**Citizens can migrate free**

Qin Daihong, deputy director of the Bureau of Policies and Regulations under the Ministry of Public Security, said the ministry has conducted extensive studies on *hukou* reform and the State Council will soon promulgate *Instructions for Unified Household Registration in Mainland China to Realize Citizens’ Right for Free Migration*. According to these instructions, the new system warrant s that citizens register their *hukou* bassed on the legal residence certificate, and their *hukou* registration place will change with their permanent residence. It means citizens’ mobility between countries and cities will not be restricted any more, which implies that “farmer” will only be an occupation instead of an official status at that time. Under this new system, the supply of educational and medical facilities, openings of jobs, pension and other social welfare are equally available to rural and urban residents. And what's more, citizens have rights of democratic management such as voting and being elected at the place where they register.
