# Supplementary material for: Discrimination against Rural-to-Urban Migrants: The Role of the Hukou System in China
Source: PLoS One. 2012 Nov 5;7(11):e46932. doi: 10.1371/journal.pone.0046932 (PMC3489849; doi:10.1371/journal.pone.0046932)
Supplement: Text S2 — Priming material of the preserving condition in English. The English version of Figure S2. (DOCX) [file pone.0046932.s004.docx]

**Text S2** Priming material of the preserving condition in English

the Chinese government announces that

**the reform of agricultural and non-agricultural *hukou* distinction needs more investigation, the current hukou system will be retained over a long period of time**

Xinhua News Agency news: The Information Office of the State Council announces in November 16^th^ that the Chinese current *hukou* system will be preserved, and Chinese Government will not make a major breakthrough on the reform of *hukou* system in the next years. The 2011 NPC (the National People’s Congress) and CPPCC (the Chinese People’s Political Consultative Conference) representatives said that it would need to control the scale of conversion from an agricultural to a non-agricultural *hukou* status for the migrants in cities. The current hukou system will be retained over a long period of time.

**Difficulty of the *hukou* system’s reform**

Qin Daihong, deputy director of the Bureau of Policies and Regulations under the Ministry of Public Security, points out that the key to *hukou* system is not the *hukou* itself, but the allocation of social welfare depending on one’s *hukou* category. One of the most important difficulties about reform on the *hukou* system is that equalization of basic public service can’t be easily implemented. It means that the government still lacks of effective methods to ensure that the migrants enjoy the equal entitlement as urban residents, such as educational and medical facilities, pension, employment opportunities and other public facilities. Take the distribution of educational resources for an example, there is huge gap between urban and rural education on many aspects like investment, promotion rate, faculty and teaching equipment, which brings the phenomenon of emigrants of the university entrance examination. In short, the problem about allocation of public resources, which cannot be resolved by simply changing the household registration calls for more funds and time. The reform on the hukou system cannot be realized within a short a period of time.
