# Supplementary material for: Discrimination against Rural-to-Urban Migrants: The Role of the Hukou System in China
Source: PLoS One. 2012 Nov 5;7(11):e46932. doi: 10.1371/journal.pone.0046932 (PMC3489849; doi:10.1371/journal.pone.0046932)
Supplement: Text S3 — Recruitment notice and resumes of the candidates. These are materials used in Study 3. Within the recruitment notice, requirements and responsibilities of a vacant job are introduced. And the resumes of two candidates, which respectively contains four sections, are showed after the notice. (DOCX) [file pone.0046932.s005.docx]

**Text S3** Recruitment notice and resumes of the candidates

**Recruitment notice for a telephone** [**customer service**](http://jobs.zhaopin.com/shanghai/CUSTOMER+SERVICE+REP_120341743251391.htm)

Requirements:

1. Female, about 25 years old, high shool or above;
2. Good command of oral Mandarin, excellent expression and communication skills;
3. Patient and service mentality, proactive, strong sense of responsibility
4. At least 2 years working experience in service industry, experience in field of customer service preferred;
5. Proficiency in Office software, fluent English would be preferred.

Responsibilities:

1. Responsible for answering calls in the telephone counseling center;
2. To provide relevant business consulting or guidance for customers;
3. To deal with customer complaints, obtaining feedback from customers;
4. Inputting the information (like conversations with customers, services offering for customers) into the database, to help the director and clients follow up the cases.

ID:JR356744417R90250001000 **zhaopin.com**

**Cui Li**

female | Unmarried | Born in 1986/01 |4 Years' Experience
Hukou: Sichuan, Lu Country, Shunhe village | agricultural category

13718242965(Mobile)

**Career Objective**

| Desired Type of Employment： | Full-time |
| --- | --- |
| Desired Position： | Customer service / sales / customer service support、Promotion specialist/Shopping guide |
| Desired Industry： | Telecom(Equipment/Operation/Value-added Service) 、Retail / Wholesale、Hotel / Restaurant |
| Desired Location： | BEIJING |
| Expected Salary(before tax)： | 2001-4000RMB/Month |
| Current Situation： | Immediately |

**Self-assessment**

Grew up in rural areas; can work hard without complaint; outgoing, honest and generous; strong sense of responsibility; have teamwork spirit; strong adaptability

**Work Experience**

| 2008/05 -- 2011/02： | Maxtv Media Company \| Telephone exchanger / front office / reception |
| --- | --- |
|  | Computer Software \| Foreign-owned \| Scale: 100-499 employees \| 1000-2000 RMB/Month |
|  | Answering and making calls, visitors reception, send and receive letters & e-mails |
| 2007/04 -- 2008/04： | Xinbaili Department Store \| Shopping guide \| Clerk / salesperson / Purchasing guide |
|  | Retail / Wholesale \| Foreign-owned \| Scale: 500-999 employees \| 1000-2000 RMB/Month |
|  | Selling goods, shop exhibition and cleanness, replenishment |
| 2006/09 – 2007/04： | Beijing Daban Housekeeping Service Company \| Housekeeping / general labor |
|  | Intermediary service \| Private-owned \| Scale: 20-99 employees \| 1000-2000 RMB/Month |
|  | Providing various housekeeping Service for employer: furnishing sweeping and cleaning, washing clothes, taking care of the elders and children, preparing lunch and supper |

**Educational Backgound**

| 2003/09 -- 2006/09： | Sichuan Luxian Occupation Technical College \| Leisure sports service and management \| Secondary specialized school |
| --- | --- |

ID:JR073882689R90250000000 **zhaopin.com**

**Jing Yang**

female | Unmarried | Born in 1985/05 |5 Years' Experience
Hukou: Beijing, Chaowai Street | non-agricultural category

13671224537(Mobile)

**Career Objective**

| Desired Type of Employment： | Full-time |
| --- | --- |
| Desired Position： | Customer service / sales / customer service support、Clerk / salesperson / Purchasing guide |
| Desired Industry： | Telecom(Equipment/Operation/Value-added Service) 、Retail / Wholesale |
| Desired Location： | BEIJING |
| Expected Salary(before tax)： | 2000-4000RMB/Month |
| Current Situation： | Immediately |

**Self-assessment**

Mild warm and generous; can work hard; good expression and communication skills; patient at work; having sense of responsibility; getting on well with colleague

**Work Experience**

| 2008/04 -- 2011/02： | China Unicom \| 1161114 \| Telephone operator |
| --- | --- |
|  | Telecom(Equipment/Operation/Value-added Service) \| State-owned \| Scale: 100-499 employees \| 1000-2000 RMB/Month |
|  | Taking service hotline, providing the route information searching service for users |
| 2007/01 -- 2008/03： | Huatangyang Huatang Store \| Clerk / salesperson / Purchasing guide |
|  | Retail / Wholesale \| Joint venture \| Less than 1000 RMB/Month |
|  | Selling products, responsible for checking goods, letting goods be out of warehouse, exhibiting, stocktaking and implementing other business activities as required |
| 2005/11 – 2006/12： | Seven-eleven(Beijing) Company Limited \| Part-time job |
|  | Retail / Wholesale \| Foreign-owned \| Scale: above 10000 employees \| Less than 1000 RMB/Month |
|  | Responsible for selling products, checking goods, letting goods be out of warehouse, exhibiting, food production, cleaning shop and other tasks as required |

**Educational Backgound**

| 2002/03 -- 2005/07： | Beijing Dongcheng District Vocational School \| Business Administration \| Secondary specialized school |
| --- | --- |
