# Supplementary material for: Discrimination against Rural-to-Urban Migrants: The Role of the Hukou System in China
Source: PLoS One. 2012 Nov 5;7(11):e46932. doi: 10.1371/journal.pone.0046932 (PMC3489849; doi:10.1371/journal.pone.0046932)
Supplement: Text S4 — Questionnaire used in the recruiting scenario. In the recruiting scenario of Study 3, participants evaluate each candidate on this questionnaire. (DOCX) [file pone.0046932.s006.docx]

**Text S4** Questionnaire used in the recruiting scenario

**Please complete the following items :**

1. The applicant’s gender: _______
2. The applicant’s *hukou* category: _______
3. The applicant’s date of birth: __________
4. The last 4 digits of the applicant’s mobile number: _________

**Please rate the applicant on the following dimensions:**

Very low Very high

1. Quality of resume 1……2……3……4……5……6……7

Very low Very high

1. Strength of past experience 1……2……3……4……5……6……7

Very low Very high

1. Applicant’s leadership skills 1……2……3……4……5……6……7

Very low Very high

1. Ability to work effectively with others in a team 1……2……3……4……5……6……7

Very low Very high

1. Ability to perform this job well 1……2……3……4……5……6……7

Very low Very high

1. Applicant’s interpersonal skills 1……2……3……4……5……6……7

Very low Very high

1. Appropriateness of applicant’s qualifications with job 1……2……3……4……5……6……7

Very low Very high

1. Ability to communicate clearly and effectively 1……2……3……4……5……6……7

Very low Very high

1. Overall impression of the applicant 1……2……3……4……5……6……7

10、If you were offering the job, how likely is it that you would hire the applicant?

Very low Very high

1……2……3……4……5……6……7

11、If the applicant were hired, how much do you think the person should earn (Yuan)?

1………2………3………4………5………6………7

1,600 2,000 2,400 2,800 3,200 3,600 4,000
